# Pure erythroid leukemia

**DOI:** 10.1002/ccr3.3056

**Published:** 2020-09-18

**Authors:** Jason C. Chen, Winston Y. Lee, Jordan K. Schaefer, Dale L. Bixby

**Affiliations:** ^1^ Division of Hematology and Medical Oncology Department of Internal Medicine University of Michigan Ann Arbor Michigan; ^2^ Department of Pathology University of Michigan Ann Arbor Michigan

**Keywords:** complex karyotype, erythroblast, erythroid leukemia, myeloid leukemia, tp53 mutation

## Abstract

The diagnosis of pure erythroid leukemia (PEL) can be challenging. Prompt identification of CD45+, CD34‐, CD71+, CD117+, and E‐cadherin+ erythroblasts is important. The differential diagnosis is broad and includes megaloblastic anemia.

## Graphical abstract

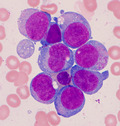

## 1 INTRODUCTION

**Figure 1:**
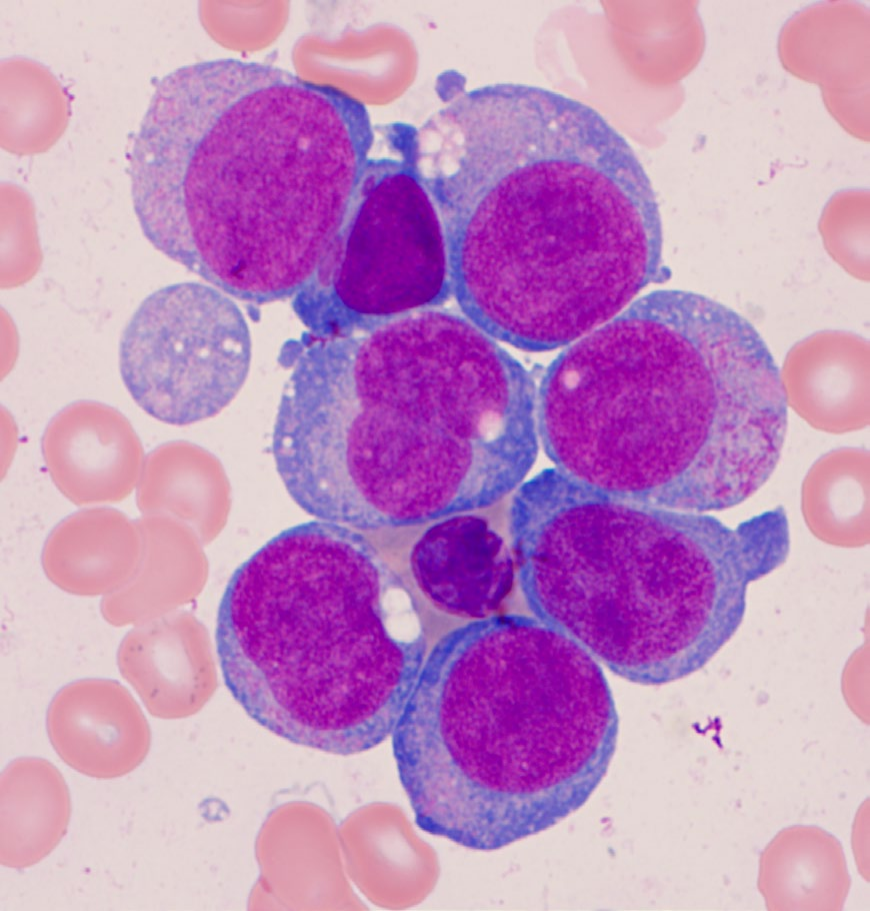
A Wright‐Giemsa stained bone marrow aspirate smear, original magnification ×1000

**Figure 2:**
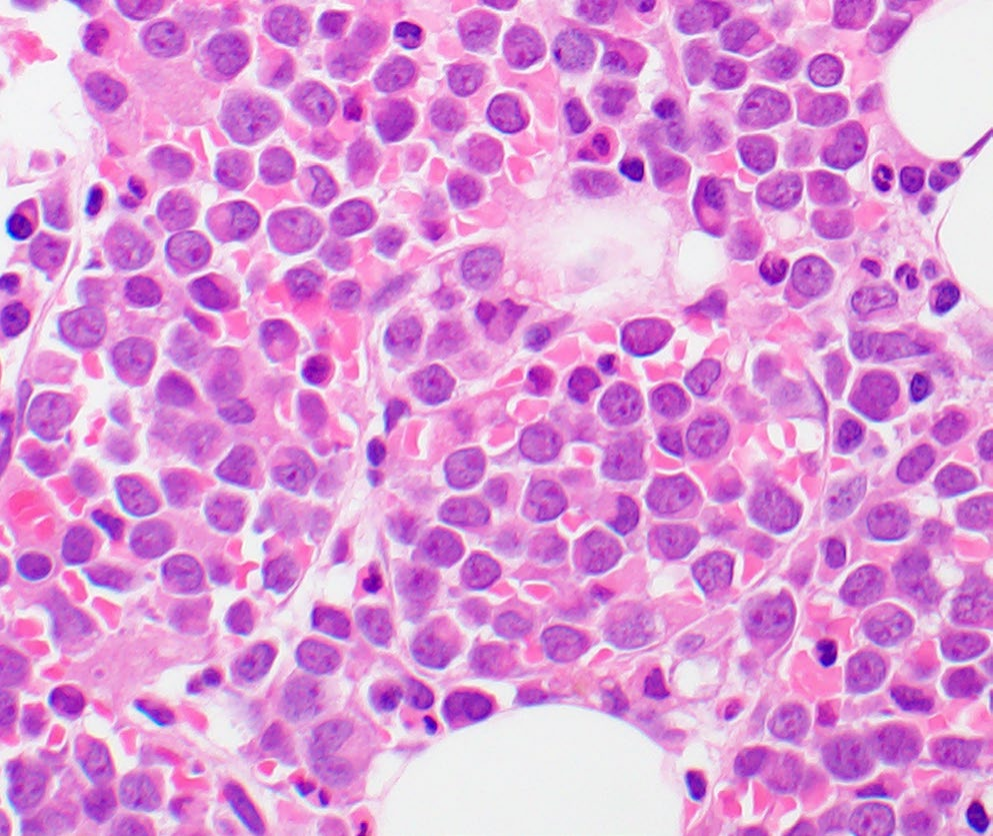
An H&E stained bone marrow core section, original magnification ×800

**Figure 3:**
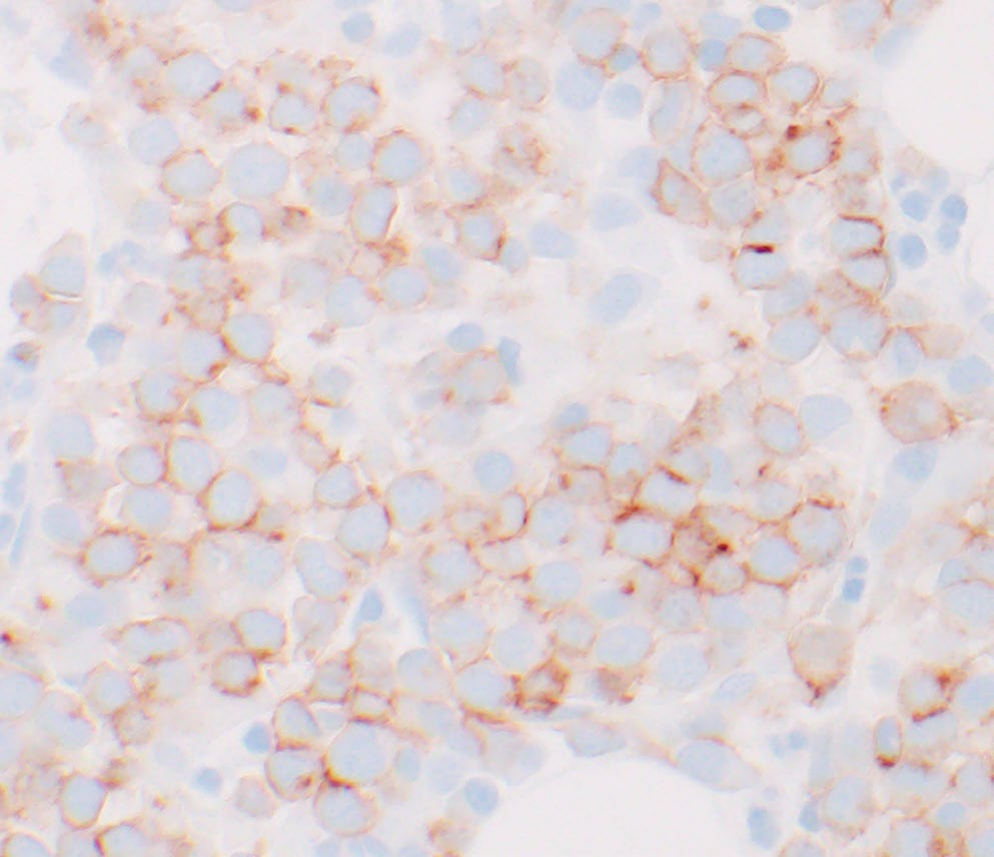
Immunohistochemical staining of a bone marrow core section for E‐cadherin, original magnification ×800

**Figure 4:**
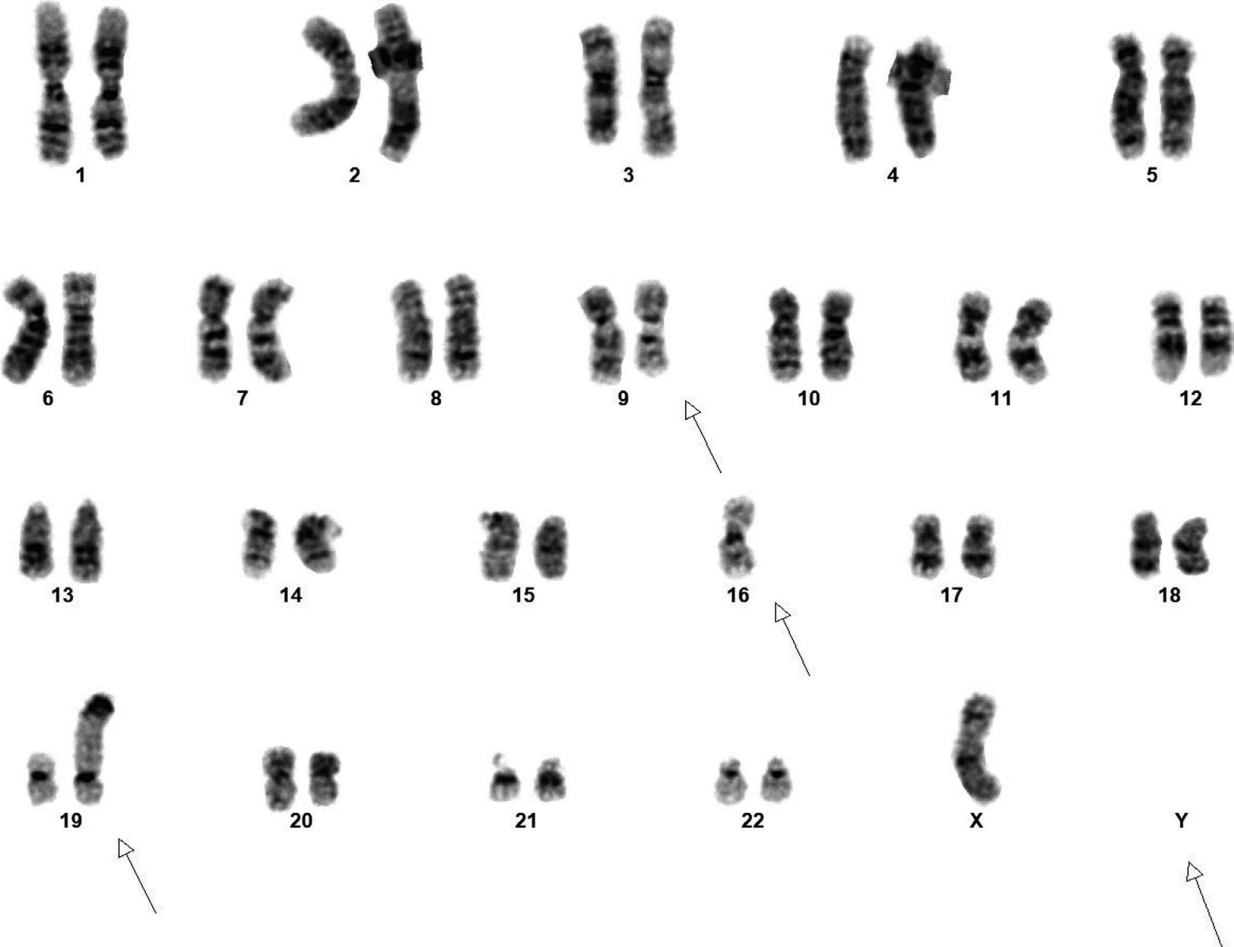
Metaphase karyotyping demonstrating a complex karyotype

A 67‐year‐old gentleman presented with pancytopenia and 2% peripheral blasts. Bone marrow biopsy showed a hypercellular marrow, 90% proerythroblasts with high nuclear/cytoplasmic ratio, fine chromatin, distinct nucleoli, and basophilic cytoplasm (Figures 1 and 2). The blasts exhibited CD45+, CD34‐, CD71+, and CD117 + by flow cytometry and E‐cadherin+ by immunohistochemical staining (Figure 3). These findings were diagnostic of pure erythroid leukemia (PEL). Cytogenetics demonstrated a complex karyotype: 44,X,‐Y,dic(9;16)(q13;p11.2),add(19)(p13)[11]/43,sl,add(15)(p13)[3]/44,sl,+6,der(12)t(12;14)(p13;q11.2),‐14[cp6]/46,XY[1] (Figure 4). Sequencing studies demonstrated loss of function TP53 mutations (TP53p.E171_R174 52.1%; TP53p.P36fs 3.3%).

PEL is defined in the 2016 WHO classification system as a neoplastic proliferation of erythroid progenitors constituting > 80% of bone marrow cellularity with ≥30% proerythroblasts and without a significant myeloblastic component.^1^ Diagnosis can be challenging due to its rarity (<1% of AML cases), CD34 negativity, and absent erythroid‐specific markers.^2^ Megaloblastic anemia related to B12/folate deficiency can have overlapping features with PEL including elevated erythroblasts. Other differential diagnoses include other acute leukemias, myelodysplastic syndrome, accelerated myeloproliferative disorders, and non‐malignant etiologies including nutritional deficiencies and myelophthisis. PEL may be therapy‐related or preceded by an antecedent myelodysplastic syndrome, is often associated with complex cytogenetics and *TP53* mutations, and has a poor prognosis with median survival of 3 months.

## CONFLICT OF INTEREST

None declared.

## AUTHOR CONTRIBUTIONS

All authors contributed to the manuscript and/or image production. JCC and WYL: assisted with manuscript writing, reviewing, and image processing. JKS and DLB: assisted with manuscript writing and reviewing. In addition, JCC and DLB: provided direct clinical care for the patient, and WYL was the pathologist that confirmed the diagnosis of pure erythroid leukemia.
